# The Electromechanical Connectome: Integrating Voltage, Mechanical Nano-Forces, and Subcellular Fluid Phase Dynamics in Human Neural Computation

**DOI:** 10.3390/ijms27042074

**Published:** 2026-02-23

**Authors:** Florin Mihail Filipoiu, Catalina-Ioana Tataru, Nicolaie Dobrin, Matei Șerban, Răzvan-Adrian Covache-Busuioc, Corneliu Toader, Mugurel Petrinel Radoi, Octavian Munteanu, Mihaly Enyedi

**Affiliations:** 1 Faculty of General Medicine, “Carol Davila” University of Medicine and Pharmacy, 050474 Bucharest, Romaniamateiserban@innbn.com (M.Ș.); octavianmunteanu@umfro.com (O.M.);; 2 Department of Anatomy, “Carol Davila” University of Medicine and Pharmacy, 050474 Bucharest, Romania; 3 Clinical Department of Ophthalmology, “Carol Davila” University of Medicine and Pharmacy, 020021 Bucharest, Romania; 4 Department of Ophthalmology, Clinical Hospital for Ophthalmological Emergencies, 010464 Bucharest, Romania; 5 “Nicolae Oblu” Clinical Hospital, 700309 Iasi, Romania; 6 Puls Med Association, 051885 Bucharest, Romania; 7 Department of Neurosurgery, “Carol Davila” University of Medicine and Pharmacy, 050474 Bucharest, Romania; 8 Department of Vascular Neurosurgery, National Institute of Neurology and Neurovascular Diseases, 077160 Bucharest, Romania

**Keywords:** electromechanical connectome, multiphysics neural computation, mechanobiology of synapses, bioelectric signaling, membrane tension and curvature, liquid–liquid phase separation, cytoplasmic rheology, metastable attractor dynamics, neurodegeneration biomarkers, AI digital twins

## Abstract

Electrophysiology, mechanobiology, and the study of soft matter within cells demonstrate increasing amounts of evidence that neuronal signaling arises from interactions between membrane potential, force, and phase. Herein, we have attempted to collect and organize the evidence for each of these areas of study into an approximate structure called the electromechanical connectome: a three-way state–space (membrane potentials, nanoscale mechanical forces, and cytoplasmic rheology, including phase-separated liquid–liquid droplets) where membrane potentials, nanoscale mechanical forces, and cytoplasmic rheology, and phase-separated liquid–liquid droplets are likely to influence one another, influencing synaptic processing, plasticity and network stability. We will also attempt to illustrate the following: how changes in electrostatic fields can be used to alter the arrangement of lipids, hydration, and dielectric microdomains, and the contact geometry between organelles and activity dependent transcription; how mechanical dynamics associated with spines, axons, and the active zone of synapses may be used to modify the energy landscape of channels, the docking and priming of vesicles, and the transport of cytoskeletons; and how viscosity corridors, along with phase-separated micro-reactors, can be used to regulate the kinetics of signaling, molecular trafficking and metabolic processes in local environments. With these connections in mind, we will propose a multiphysical attractor model in which cognition is the result of navigating through metastable manifolds, while neurodegenerative disease may be a result of the progressive loss of electromechanical coherence, phase boundary control and energetic flexibility. Finally, we will present testable hypotheses and use AI-enabled digital twin methods to potentially quantify the early deformation of manifolds and provide precision biomarkers and therapeutic options.

## 1. Introduction: Revealing the Hidden Biophysics Beneath Neural Computation

While the study of neural computation has historically been based on models centered on membrane excitability, ion channel kinetics, synaptic transmission, and circuit structure, it is becoming increasingly clear that there are many additional biophysical substrates that also play important roles in how neural computations occur. In particular, the work described here indicates that while electrical and synaptic mechanisms are necessary for neural computations to occur, they are only one aspect of the biophysical substrate that supports computation in the nervous system [1,2]. This study finds that neurons exist in a unique microenvironment that includes dynamic membranes, force-generating cytoskeletal networks, and intracellular fluids whose physical properties vary both geographically and temporally. Furthermore, advances in high-resolution imaging, soft matter biophysics, and molecular neurochemistry show that electrical activity is continually interacting with mechanical nano-forces and fluid phase processes to impact computation in ways that have not been accounted for by previous models of neural computation [3].

The electromechanical connectome model of neural processing does not replace or supplant traditional models of neural processing. Rather, it extends the list of measurable physical parameters interacting with the electrical activity of neurons [4]. Therefore, these additional measurable physical parameters comprise the following: the fields of mechanical tension and curvature at the nanoscale; the nanoscale physical structure of the fluids within neurons (for example, the rheological properties of the cytoskeleton, and the liquid–liquid condensate formation resulting from the phase separation of liquids); and the phenomenon that when a neuron is encoding information at two scales of time, (i) quickly through electrical activity, and (ii) at a slower pace because of the physical characteristics of the neuron (e.g., viscosity gradients, the condensed materials’ properties, and the mechanically supported architecture of the neuron), the neuron will encode information at both of those timescales [5]. This perspective is compatible with the authors’ concept of the paper that neural computation arises from the entire physical state of the neuron and its environment, and not just from the electrical activity of the neuron [6].

Therefore, the electromechanical connectome is compatible with all of the fundamental mechanisms of neural processing (membrane excitability, synaptic transmission, and interneuronal connections). The electromechanical connectome also presents a new way to increase the number of measurable physical parameters in the interactions with electrical activity while processing neural signals. As such, the electromechanical connectome may be able to expand the measurable space of possible neural computation by adding new measurable variables in addition to the fundamental processes of neural processing [7].

For decades, membrane potential was viewed as an electrical variable that influenced the kinetic gating of sodium, potassium, and calcium channels. However, it is now apparent that membrane potential can shape structural and molecular states over multiple timescales. For example, subthreshold membrane voltage fluctuations and depolarizing dendritic plateau potentials cause significant changes in the conformational landscape of Cav1.2, Nav1.6, HCN1, and several other types of ion channels by changing the local electric field experienced by the voltage-sensing domains of these channels [8]. Additionally, voltage-dependent calcium influx causes the activation of several types of protein kinase, including calmodulin, CaMKII, calcineurin, and members of the MAPK family. The activation of these kinases leads to the regulation of histone acetyltransferases, histone deacetylases, chromatin remodeling factors, and methyl transferases, ultimately leading to changes in nucleosomal spacing, loop dynamics between enhancers and promoters, and the binding of transcription factors to specific genomic locations associated with neural activity [9]. Therefore, the data indicate that electrical activity can modulate gene expression and the physical structure of chromatin through multiple independent pathways. Furthermore, mechanical forces can modulate neural computation with a similar spatial resolution as electrical activity. The plasma membrane functions as a viscoelastic interface that can be dynamically modified by exocytosis, actin polymerization, deformation of the spectrin–actin lattice, and interactions with external matrix proteins. The modulation of the plasma membrane’s viscoelasticity can alter the gating characteristics of mechanically sensitive ion channels such as PIEZO1/2, TREK-1, TRAAK, and TRPV4, and the ASIC family of channels [10]. Each of these mechanically gated channels exhibit tension-dependent conductance and contribute to the subthreshold modulation of the electrical excitability of neurons. Additionally, changes in mechanical forces at presynaptic terminals can alter the geometric relationships between vesicles and the active zone, and therefore can modulate the rate of vesicle release. Rapid cycles of actin polymerization and depolymerization in dendritic spines modify local stiffness, neck resistance, diffusion barriers, and receptor trafficking. These changes in spine morphology can create signaling nanodomains containing populations of AMPA and NMDA receptors, MAGUK family scaffolding proteins, CaMKII holozymes, and Ras-GTP microdomains, influencing the amplitude, timing, and integration of synaptic responses [11].

Finally, the fluid phase behavior of the cytoplasm represents a third layer of modulation that impacts neural computation. The cytoplasm is extremely dense and highly crowded, with large numbers of macromolecules resulting in non-Newtonian rheological behavior and compartment-specific viscosity gradients. Studies utilizing fluorescence recovery after photobleaching (FRAP), genetically encoded viscosity probes, and active microrheology indicate that the viscosity and viscoelastic relaxation times of the cytoplasm can be significantly different among the various compartments of the cell (somatic, dendritic, and axonal) and can vary depending upon the metabolic state of the cell, age, and injury status [12]. These differences in viscosity can influence the movement of organelles (mitochondria, lysosomes, endosomes, etc.) and ribonucleoprotein particles (RNA granules) along microtubule tracks mediated by motor proteins (kinesin-1, kinesin-3, dynein, and myosin-V). Liquid–liquid phase-separated (LLPS) condensates contain many signaling components including those found in presynaptic active zones (RIM and Liprin-α), postsynaptic densities (PSD-95, SynGAP, and Shank), RNA-binding proteins (FUS, TDP-43, and hnRNPA1), and transcriptional regulators within the nucleus [13]. These phase-separated condensates create microenvironment with altered chemical potentials, reaction rates, and ion-buffering properties, allowing for slow changes in the computational state of the neuron that complement fast electrical signaling. Increasing amounts of data suggest that electrical activity, mechanical forces, and fluid phase behavior interact with each other in a coordinated manner primarily through the same physical constraints. For example, changes in the membrane potential can alter the membrane tension through changes in lipid packing and cytoskeletal anchorage [14]. Similarly, mechanical deformation can influence the distribution and conformational equilibrium of ion channels, thus modifying local electrical states. Finally, changes in cytoplasmic viscosity or the material properties of phase-separated condensates can influence the diffusion-limited processes that govern calcium buffering, kinase/phosphatase activation, metabolic gradients, and the access of signaling molecules to synaptic or nuclear compartments. The cooperative interaction of electrical activity, mechanical forces, and fluid phase behavior suggests that neural computation occurs due to the complete physical state of the neuron, including electrical potentials, mechanical stress fields, and phase state organization [15].

Recent technological advancements have enabled researchers to measure the interactions of electrical activity, mechanical nano-forces, and fluid phase behavior in real-time with nanometer-to-micrometer spatial resolution. Techniques that enable these measurements include high-speed atomic force microscopy, interferometric scattering microscopy, correlative light and electron microscopy, lattice light sheet imaging, FRET-based tension sensors, and phase-sensitive fluorescent reporters [16]. While these technologies are rapidly advancing, they provide a new understanding of the neuron as a soft, heterogeneous material system where electrical excitability, mechanical stability, and fluid phase organization all contribute to signal propagation, synaptic reliability, and long-term adaptation [17].

In this review we will attempt to synthesize the recent findings regarding how electrical activity, mechanical nano-forces, and fluid phase behavior interact to support neural computation. It is not our goal to present definitive conclusions but rather to develop a unifying framework that incorporates results from multiple areas of research, including electrophysiology, molecular imaging, cryogenic structural biology, rheology, and single-molecule biophysics. To accomplish this goal, we will use a wide range of primary studies that have been published in the last two years to provide a comprehensive view of the developing electromechanical connectome.

This is a conceptual analysis of how structures have evolved and operate in relation to their design and performance capabilities; this is a methodologically based scoping synthesis which is not a systematic review. This literature review used PubMed/Medline and Web of Science, and targeted searches in Google Scholar, since much of the research in biophysics is indexed under biomedical indexes, although the research could also have been indexed under biomedical indexes. It searched back and forth through citations of the most relevant articles in methodologies and fields related to cross-disciplinary interfaces and interfacial regions (membrane electromechanics (mechanical properties of membranes (tension, curvature, lateral pressure, and lipid packing), S4 domains for voltage sensing, membrane dipoles, and hydration shells), mechanotransduction (ion channels which are mechanically gated (PIEZO, K2P/TREK/TRAAK, TRP, and ASIC)) and cytoskeletal mechanics (stability/stiffness of actin-spectrin lattices and microtubules), the architecture of the substrate at the nanoscale (postsynaptic density, axon initial segment, synaptic nanodomain, and synaptic active zone)), the states of intracellular materials (viscoelasticity/rheology/microrheology/fluorescence recovery after photobleaching), phase biology (biomolecular condensation, liquid–liquid phase separation, gelation, and RNA granules), formalisms which provide the tools to combine these domains (reaction–diffusion and attractor dynamics), and into disease (AD/PD/FTD/ALS). This study will be focused primarily upon quantitative analyses and studies employing quantitatively based models to measure, determine, or modify a defined physical property (e.g., tension, curvature, stiffness, viscosity, etc.) of an intracellular component and relate it to physiological, signaling, trafficking, and/or synaptic output; this study will be limited to studies that described phenomena but did not describe a cross-domain mechanistic relationship, and will consist of primarily foundational studies, in order to understand the basic principles of cross-domain interactions. The large number of citations indicates the convergence of new technologies (live-cell imaging, multiphysics modeling, phase-sensitive methods, structural methods, and rheological methods) that now enable researchers to measure and compare the effects of electrical/mechanical/phase coupling at biological scales.

The subsequent sections will construct this view of the electromechanical connectome. Section 2 will describe the biophysical foundation of electromechanical interactions, including energy landscapes, membrane mechanics, cytoskeletal force generation, and intracellular rheology. The subsequent sections will examine the regulation of genomic and structural states by electrical activity, the role of mechanical forces in synaptic and dendritic compartments, the role of phase transitions and condensate behavior in neural computation, and the development of integrated computational models that account for these processes. We will conclude by discussing how disruptions of the electromechanical connectome may lead to neurological disorders and by describing possible future avenues for studying the physical basis of brain function.

## 2. Biophysical Foundations of the Electromechanical Connectome

### 2.1. Voltage Sensing, Gating Energetics, and Ionic Microenvironments

The voltage-gated ion channel is able to take a change in the membrane potential and turn it into a change in the conformation of the S4 helix (of the voltage-sensing domain) which is inside the channel; recent studies using cryo-EM and atomic-level simulation have demonstrated that there are no single motions of the S4 helix as the channel gates, but instead a series of metastable intermediates [18,19]. These intermediates each exist at different levels of contact with the lipid head groups, the membrane dipoles, and the hydration shell; therefore, the movement of charge associated with the channel gate does not occur solely through the motion of the positive charges, but also by the reorganization of the local electrostatic field [20].

Voltage-gated channel operation is much more closely tied to the physical characteristics of the membrane than was previously assumed. For example, lipids such as PIP2, DAG, and PS create anisotropic electric fields that both affect the amount of gating charge and the energy barrier for moving between open, closed and inactivated states. The surrounding water layer adds another level of complexity to the process. Ultrafast infrared spectroscopy indicates that the hydration networks around the channel rapidly reorganize during channel transitions and influence proton mobility and local dielectric constants [21]. This causes the rate of gating to vary over microdomains, which explains why the same isoform of an ion channel will exhibit different operating modes in the axonal initial segment, distal dendrite, and presynaptic bouton. Local ionic microdomains further influence electrical behavior [22]. Concentration gradients of Ca^2+^, generated by Cav2.1 and Cav2.2 channels, can be very steep and extend only tens of nm, allowing for the highly specific activation of the machinery required for vesicle fusion or activation of Ca^2+^-sensitive enzymes. These gradients, in turn, generate transient surface potentials that slightly depolarize or hyperpolarize adjacent membrane patches and thereby influence the likelihood of neighboring channels opening. Similar types of microdomains that contain high levels of K^+^ efflux can generate changes in osmotic gradients that will cause changes in the water flux and thus changes in membrane tension [23].

This multilayered electrical substrate forms the dynamic background against which mechanical and fluid phase interactions take place to regulate the flow of information in the neuron, forming a distributed computational system based on the collective behavior of gating energetics, lipid microstructure, and ionic topology [24].

### 2.2. Mechanical Signal Propagation Across Membranes, Cytoskeleton, and Adhesion Complexes

The mechanical forces in neurons arise from the interaction of cytoskeletal polymers, membrane curvature and the external anchor points. The mechanical forces generated in the cell propagate through several pathways that display the properties of the materials used in composite engineering systems. Actin filaments produce forces in the order of pN through ATP-driven polymerization and myosin II-dependent contraction. Studies of single-molecule force spectroscopy have shown that individual actin–cofilin units exist in two different states; one that is relatively rigid and one that is more flexible depending on their phosphorylation and oxidation status, indicating that neurons may modulate the stiffness of their actin filaments on sub-second timescales during signaling [25].

Microtubules behave in a different manner and serve as stiff, load-bearing conduit that depend on the nucleotide state of tubulin and the growing number of post-translational modifications for their stability. Gradients of polyglutamylation and detyrosination along dendrites and axons appear to produce mechanical anisotropy that influences the behavior of motor proteins, the transport of organelles and the transmission of deformation [26]. The stiffness of microtubules is further influenced by structural proteins such as MAP6, which protect microtubules from cold and mechanical stress and allow for the long-term persistence of deformation signals. Spectrin lattices, especially the αII–βII spectrin lattice that occurs in the axon, provide a mechanism for elastic support that protects the axolemma during high-frequency firing [27,28]. Atomic force microscopy has shown that these lattices are capable of reversible extension and compression, and distribute mechanical stresses across nodes of Ranvier and the initial segments. Spectrin lattices also interact with ankyrin-G and transmit mechanical deformations to the organization of voltage-gated sodium channels, establishing a direct connection between membrane mechanics and electrical excitability [29].

Information about mechanical forces is also transmitted across the membrane through mechanotransduction complexes that include cadherins, integrins, talin and vinculin. Talin molecules unfold when subjected to force and expose new binding sites that can increase the strength of adhesion complexes and modulate subsequent actin remodeling [30]. Protein-level events that integrate extracellular stiffness, shear forces or curvature changes with intracellular cytoskeletal dynamics occur in regions of high membrane curvature and recruit BAR-domain proteins, which convert geometric information into biochemical signals that influence endocytosis, spine remodeling or presynaptic vesicle cycling [31].

Therefore, mechanical forces propagate through a network of polymeric and membrane-associated structures that operate at different force and timescales. These pathways continuously modulate neuronal morphology, channel clustering and synaptic organization, and form a mechanical substrate that interacts with electrical and biochemical signals to modulate the computational behavior of the neuron [32].

### 2.3. Intracellular Rheology, Phase-State Constraints, and Soft Matter Regulation of Neural Signaling

The cytoplasm of the neuron behaves as a dynamic soft matter environment and its rheological properties determine how signals propagate throughout the cell. Instead of being a simple aqueous solution, the cytoplasm of the neuron behaves as a viscoelastic substance whose properties are determined by the compartmentalization of the cell and the physiological condition [33]. Optical trapping and active microrheology experiments have shown that the cytoplasm of the neuron relaxes in the millisecond to second range of timescales, which reflects the contributions of polymer entanglements, organelle crowding and transient binding interactions to the cytoplasmic properties. These properties constrain the diffusion of proteins, metabolites and second messengers and impose a spatial structure on biochemical reaction networks [34].

The crowding of molecules caused by the high concentrations of proteins, ribonucleoprotein complexes and cytoskeletal elements alter the thermodynamics of binding events by stabilizing intermediate states and promoting multi-valent interactions. Crowding creates a condition favorable for the formation of phase-separated condensates consisting of intrinsically disordered regions of proteins such as PSD-95, SynGAP, RIM or FUS. Condensates represent reaction crucibles whose viscosity, internal mesh size and interfacial tension modulate reaction rates and molecular turnover [35]. They establish what type of behavior (liquid drop, gel-like, etc.) these condensates will exhibit under stress and aging. Gradients of rheological properties along the length of the dendrite and axon influence the transport of organelles and the distribution of ATP and calcium buffering capacity, generating spatially heterogeneous metabolic environments. The transport of endosomes, lysosomes and RNA granules also exhibit compartment-specific transport dynamics, which influence their ability to access synapses or the nucleus [36].

Structured hydration layers surrounding lipids, filaments and aggregates also contribute to the nuance of the properties of the cytoplasm. Hydration layers modulate local dielectric constants, ion mobility and proton transfer, and as a result influence the rates of enzymatic reactions, the stability of protein complexes and the occurrence of phase transitions. Changes in osmotic pressure or ionic strength can also induce changes in the boundaries of condensates and induce fusion, fission or dissolution events that reorganize signaling landscapes [37].

In this way, the rheological properties of the cytoplasm and the constraints imposed by phase state organization provide additional levels of modulation of the electrical and mechanical behaviors of the neuron. Changes in viscosity can either accelerate or impede signaling cascades, affect the speed of intracellular calcium wave propagation and modulate the coupling between electrical events at the membrane and genomic responses. Therefore, rheological properties and phase state organization represent slow, modulable parameters that shape the outcome of computational processes and define the biophysical environment in which electrical and mechanical processes operate [38,39]. Table 1 intends to condense the core biophysical elements that underpin electromechanical signaling in neurons, outlining how electrical, mechanical, and soft matter processes converge to shape the operational logic of the connectome.

## 3. Voltage as a Structural and Genomic Signal

### 3.1. Activity-Dependent Chromatin Remodeling and Electrically Driven Nuclear Reorganization

Depolarization triggers a variety of molecular events that lead to the reorganization of the chromatin architecture in a highly spatially specific manner. Using single-nucleus multi-omic profiling and live cell imaging, researchers have demonstrated that depolarization rapidly produces fast Ca^2+^ microdomains at nuclear pore invaginations rich in IP_3_ receptors and nucleoplasmic reticulum extensions sensitive to voltage. These Ca^2+^ microdomains activate a series of nuclear kinases, including CaMKIV, MSK1 and CaMKK2, which in turn phosphorylate histone H3 on serine 10 and recruit bromodomain-containing proteins to enhancer regions to produce a rapid activation of the gene expression programs involved in the early response to electrical activity [47].

Recent high-resolution studies of chromatin conformations have identified that electrical activity rapidly produces cohesion-mediated loop extrusion and reorganizes 3D chromatin topology within minutes of membrane depolarization. The production of loops brings together long-range regulatory elements and early-response genes, enabling short-lived transcriptional bursts representing the frequency and intensity of the most recent electrical activity [48,49]. However, this structural response is not uniform throughout the genome, and different cell types show unique regulatory topologies linked to their electrical activity. For example, perisomatic interneurons, layer V pyramidal neurons and cerebellar Purkinje cells each display unique regulatory topologies that suggest a cell-type-specific relationship between electrical activity and higher-order genomic folding. In addition, depolarization produces the rapid reorganization of nuclear condensates. The voltage-dependent reorganization of mediator clusters and Pol II super-assemblies alter the viscosity and internal mobility of the condensates [50]. The increased phosphorylation of the C-terminal domain of RNA polymerase II promotes the structural fluidisation of the condensates, affecting both the size and duration of transcriptional bursts, and thereby linking electrophysiological states to transcriptional kinetic behavior via the material properties of the condensates [51].

The collective data demonstrate that electrical activity is an organizing force that alters the nuclear topology, chromatin accessibility and transcriptional condensate behavior in ways consistent with the computational needs of the neuron [52].

### 3.2. Electrogenomic Pathways Integrating Membrane Voltage with Metabolic, Translational, and Proteostatic Architecture

Voltage influences processes extending well beyond gene expression: it synchronizes the metabolic and translational processes defining the organization and function of the cell. Depolarization also modulates the mitochondrial Ca^2+^ uptake through the voltage-dependent recruitment of the MCU-MICU1/2 complex, thereby modulating the redox balance and activity of NAD^+^-dependent dehydrogenases. As a consequence, this resets mitochondrial substrate utilization and modulates ATP/ADP ratios in subcellular microdomains, thereby regulating processes such as actin polymerization, lipid modification and localized protein synthesis [53]. Electrical activity also regulates the morphology of the Endoplasmic Reticulum (ER). The voltage-dependent Ca^2+^ release from ER stores reconfigures the tubular ER networks through membrane fusion and curvature-sensitive reticulon scaffold regulation mediated by atlastin [54]. These geometric changes modify the ER–mitochondria and ER–plasma membrane contact sites, thereby modulating lipid exchange, phosphoinositide metabolism and the localization of Ca^2+^-signaling nanodomains. Thus, voltage-dependent modifications to the geometry of the ER regulate how excitatory and inhibitory signals are spatially integrated through the generation of Ca^2+^ waves and the localization of biosynthetic machinery [55].

Voltage states also regulate the localization and translation of RNAs through the phospho-regulation of RNA-binding proteins containing low-complexity domains, such as FMRP, Staufen, hnRNPA2B1 and TDP-43. Electrical activity also modulates the phase behavior of ribonucleoprotein (RNP) granules, thereby modifying their viscosity, surface tension and degree of cross-linking [56]. These changes regulate whether mRNAs are retained in translationally inactive condensates or are made accessible for translation at synaptic sites. Finally, the voltage-dependent regulation of ribosomal protein phosphorylation further adjusts the elongation rate of translation and links electrical activity to proteome remodeling [57].

Finally, electrical activity also regulates proteostasis. Depolarization regulates the activity of Ca^2+^-regulated chaperones and proteases, including calpains, mitochondrial Lon protease and cytoplasmic HSP70/90 cycles. Voltage-dependent proteostatic pathways regulate the degradation of ion channels, synaptic proteins and cytoskeletal proteins, thereby regulating the structural and signaling capabilities of the neuron based upon its electrical history. Thus, membrane voltage generates a cellular network integrating metabolic flow, membrane trafficking, translational output and protein quality control with electrical activity patterns. This electrogenic coupling enables the neuron to organize its biochemical and structural landscapes to meet the computational demands of the neuron [58].

### 3.3. Electrical Regulation of Subcellular Geometry, Organelle Microdomains, and Spatial Signaling Architecture

Voltage states do not merely regulate molecular processes, but also modulate the physical organization of subcellular structures. In axons, the firing frequency modulates the distance between mitochondria through Ca^2+^ regulation of the Miro/TRAK transport complex. High-frequency activity increases the presence of mitochondria adjacent to nodes of Ranvier and active boutons, thereby enhancing the availability of ATP, which is required for the functioning of Na^+^/K^+^-ATPase, vesicle cycling and local Ca^2+^ buffering. The redistribution of mitochondria creates a spatial energy landscape that dynamically responds to the electrical activity of the axon [59].

Similarly, dendritic signaling is modulated by the voltage-dependent reorganization of microtubules and actin filaments. Chronic depolarization activates cofilin phosphatases and formin-family nucleators, producing changes in filament turnover and mechanical stiffness that modify the resistance of the spine neck, the properties of the dendritic cables and the attenuation of the signal [60]. Therefore, the electrodynamical development of dendritic arbors develops concurrently with the morphological development of dendritic arbors through the modulation of these structural parameters [61].

Finally, the voltage-dependent modification of endosomal and lysosomal trafficking modulates the rates of receptor recycling, degradation and synaptic protein turnover. Voltage-dependent Ca^2+^ regulation of rab-GTPases and their downstream effectors modulate the directionality of transport, the rate of vesicle fusion and the identity of the compartment. Therefore, the voltage-dependent modification of the stoichiometries of AMPA, NMDA and GABA receptor populations at the synapse and the spatial tuning of excitatory and inhibitory inputs at the dendrite depend on the geometry of the ER and the contacts between the ER and other organelles [62]. The voltage-dependent modification of the lipid bilayer affects the packing density of lipids, the acyl chain ordering and the distribution of cholesterol nanodomains. The voltage-dependent modification of phospholipase C and phosphatidylinositol kinases modulate the amounts of PIP_2_, PIP_3_ and DAG, and therefore modulate the recruitment and stability of scaffolding molecules and channels. Therefore, the geometry of the microdomains of the plasma membrane is altered, and this affects the excitability of the cell and the spatial organization of receptor and channel clusters [63].

**Figure 1:**
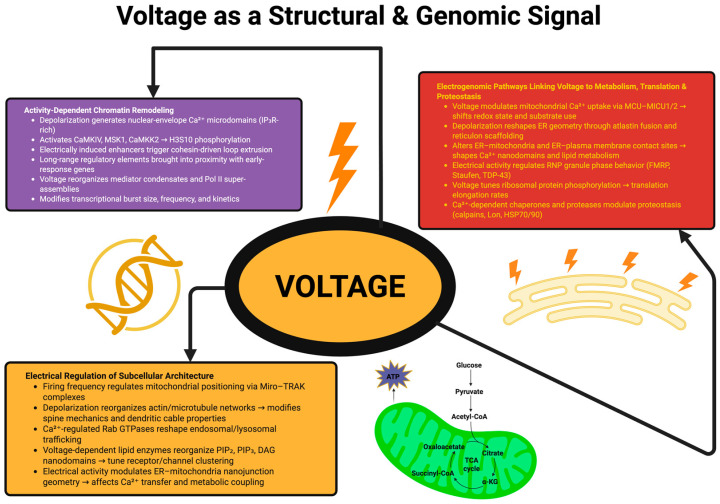
Illustrates how membrane voltage serves as an organizational variable that alters both nuclear architecture and subcellular organization and regulates metabolic processes within the cell; the central focus of the figure shows voltage as the major integrating force connecting excitability with regulatory processes in cells.

Electrical activity also affects the geometry of the nanojunctions formed between organelles, such as ER–mitochondria or ER–plasma membrane contact sites. The voltage-dependent modification of Ca^2+^ and lipid fluxes modulates the tethers formed between organelles by proteins such as STIM1/2, VAPB, MFN2 and ORP-family lipid transporters, and thereby modulate the widths and continuities of the junctions. Since these junctions are essential for the transfer of Ca^2+^, the coordination of metabolisms and the exchange of lipids between organelles, the modification of the geometry of the junctions directly affects the fidelity and timing of the intracellular signaling [64]. Thus, electrical activity is a global organizer of subcellular architecture. Electrical activity creates dynamic changes in the geometry of organelles, the mechanical properties of the cytoskeleton, the topology of membranes and the geometry of signaling microdomains that directly modulate the spatial logic providing the basis for information processing in individual neurons [65]. Figure 1 aims to summarize how membrane voltage acts as a unifying physical signal that extends beyond excitability, reorganizing nuclear structure, metabolic programs, and subcellular architecture.

## 4. Mechanical Nano-Computation in Synapses, Dendrites, and Axons

### 4.1. Mechanosensitive Channel Logic and Tension-Encoded Modulation of Excitability

The mechanical deformation of a cell’s membrane introduces new computational variables into neuronal signaling via the modification of the way the bilayer deformation is interpreted by the ion channels; the membrane deformation is due to mechanical forces. In addition to being sensitive to changes in membrane tension, mechanosensitive channels, e.g., Piezo1/2, are sensitive to changes in the degree of deformation of the membrane bilayer; the Piezo channels’ conformational flexibility is directly correlated with the membrane tension [66]. Their transmembrane architecture is a curved, “blade-like” structure which dilates along the plane of the membrane as it becomes increasingly tense, thus yielding a series of graded conductance states rather than simply binary transitions. Using this type of mechanism, neurons can sense nanometer-scale deformations of the membrane caused by synaptic activity, and fluctuations in the osmotically induced deformations of the cell membrane or by the deformation of the cell membrane associated with its cytoskeleton. Ion channels known as two pore domain (K2P), i.e., TREK-1, TREK-2, TRAAK and other related K2P channels, integrate mechanical data from the surrounding environment by means of lipid–protein coupling; thus, the mechanical deformation of the membrane causes changes in the position of pore-lining helices of the ion channels and consequently modulates potassium leak currents [67]. These mechanically gated currents contribute to the stabilization of the membrane potential by responding to the deformation of the membrane, and represent a fast feedback loop that modifies the excitability of the neuron independently of the excitability-modifying effects of classical, voltage-dependent ion channels [68].

Unlike each other, acid-sensing ion channels (ASICs) and TRPV4 use different mechanisms to respond to deformation of the membrane. ASICs are subject to protonation-dependent gating which is increased by membrane compression; conversely, TRPV4 responds to membrane deformation via lipid-dependent allosteric transitions. Since the mechanical deformation of the membrane occurs over short distances of membranes without relying on ionic diffusion, these mechanically gated contributions allow for the creation of spatially heterogeneous micro-circuits of excitability. These tension-dependent modifications enable the encoding of mechanical events, at the millisecond timescale, into precise electrical responses, thereby providing an additional dimension of computation in addition to voltage-dependent signaling [69].

### 4.2. Spine and Dendritic Mechanics as Distributed Analog Computation Modules

The spines of dendrites function as flexible computation modules whose mechanical properties determine both synaptic efficacy and the summation of signals. High-speed imaging and nano-indentation have shown that the mechanical properties of spine heads vary depending on the location and therefore have a variety of mechanical compliances, which are determined by the localized actin–cofilin dynamics, tropomyosin distribution, and spectrin-based cross-linking [70,71]. Thus, the rate of the deformation of spines during synaptic stimulation will be dependent on the mechanical compliance of the spine head, and will alter the ionic micro-geometry and the effective electrical resistance of the spine neck. Both of these will modify the magnitude of the voltage transients reaching the dendritic shaft and modify the integrative functions of the neuron without modifying the density of channels [72].

In addition to modifying the integrative functions of the neuron, the mechanical deformation of spines also affects the organization of receptors at the synapse. Curvature-sensitive proteins, e.g., endophilin and amphiphysin, tend to accumulate at the locations of the spine where there is temporary bending, and thereby stabilize AMPAR receptor clusters by creating localized lipid defects that facilitate receptor incorporation. This mechanically induced receptor organization influences the kinetics of postsynaptic currents and facilitates the alignment of receptor clusters with the site of presynaptic release [73].

Mechanical cues also affect biochemical signaling within spines. The strain on phosphoinositide-rich nanodomains will alter the accessibility of PIP_2_ to PLCβ and PI3K, and therefore affect the generation of DAG and PIP_3_. These biochemical reactions occur at rates that are influenced by the local mechanical stiffness and therefore generate spatially restricted biochemical states whose duration is dependent on actin turnover and the mechanical properties of the membrane rather than solely the enzymatic properties of the reaction [74]. A similar hierarchical organization of mechanical anisotropies is found in the dendritic branch; mechanical anisotropies are created by the density of actin–microtubule interactions, the density of microtubules, the presence of microtubule bundles and the density of MAP-dependent cross-links. These mechanical anisotropies create structural gradients that affect the attenuation and spread of synaptic signals. Thus, through the described mechanisms, dendrites and spines convert electrical, biochemical, and mechanical information into a continuous, analog computational process that enables the neuron to modulate its sensitivity to stimuli based on its local physical state [75]. The goal of Table 2 is to provide a systematic overview of how mechanical forces operate as computational variables in neurons.

### 4.3. Presynaptic Mechanotransduction in Vesicle Positioning, Priming, and Fusion

The active zones of presynaptic terminals use mechanical parameters to precisely control the probability of neurotransmitter release. Scaffolding proteins such as ELKS, RIM, and Munc13 produce localized tension fields across the presynaptic membrane due to the elastic properties of the protein scaffold; these tension fields alter the curvature of docking sites and the energy required for SNARE assembly, determining if the vesicles are positioned in intermediate states tethered to the presynaptic membrane or in fusion-ready positions [76].

Cryo-electron microscopy has shown that the interfaces between vesicles docked at mechanically favorable sites are flattened and exhibit minimal membrane separation, facilitating the zippering of SNARE complexes and rapid Ca^2+^-dependent fusion. Vesicles that encounter regions of altered curvature show delayed progression of the priming intermediates, establishing a mechanical basis for the heterogeneity of release probability even within single active zones [77]. The additional mechanical modulation of vesicle dynamics is provided by cortical mechanics. The actin–synapsin matrix creates a viscoelastic barrier that limits vesicle mobility and affects the replenishment of the readily releasable pool of vesicles. The softening of the matrix during high-frequency firing enhances vesicle transit and supports sustained neurotransmission, while increased stiffness during metabolic compromise reduces vesicle recruitment and contributes to synaptic fatigue [78].

Finally, endocytosis is critically dependent on membrane tension and curvature. Clathrin-mediated vesicle retrieval involves the coordinated action of BAR-domain proteins that generate the curvature of the membrane and dynamin whose constriction efficiency is proportional to the bending energy present in the membrane. The mechanical properties of the presynaptic terminal modulate the kinetics of pit formation and vesicle scission and thus synchronize the recovery of endocytosis with the mechanical context established by preceding activity [79]. Axons contribute additional mechanical modulation of the presynaptic terminal through their spectrin-based periodic cytoskeleton. The spectrin-based lattice distributes longitudinal strains along the axon, preserves action potential fidelity during repetitive firing, and maintains the structural conditions necessary for reliable vesicle release. Thus, mechanical feedback within the axonal shaft ensures that presynaptic terminals operate in an optimal range of deformation, coordinating mechanical integrity with synaptic function [80]. Section 4 identifies mechanical forces as independent computational variables and describes the mechanisms by which mechanically sensitive channels, deformable dendritic spines and mechanically structured presynaptic terminals cooperatively form a distributed mechanical computing architecture. Through these mechanisms, mechanical forces become a fourth layer of processing in the electromechanical connectome, in addition to the electrical, chemical and structural layers.

## 5. Intracellular Fluid Phase and Condensate Logic in Neural Signaling

### 5.1. Cytoplasmic Rheology, Non-Equilibrium Viscosity Fields, and Diffusion-Limited Computation

The cytoplasm of neurons has been shown to be a non-equilibrium soft matter that fluctuates in terms of physical properties in response to metabolic and electrical activity. In other words, it does not behave uniformly throughout the cell. Instead, the cytoplasm contains dynamic viscosity fields that are influenced by ATP availability, macromolecular crowding, local pH levels, oxidative gradients and the transient assembly of polymer networks. Recent advances in interferometric scattering microscopy demonstrate that the viscosity fields of the cytoplasm are continually being modified during the electrical activity of the cell, thus producing spatial and temporal patterns that distinguish one type of neuron from another [81].

These non-equilibrium rheologic states impart a directional bias to the diffusion of signaling molecules. Simulations and tracking studies conducted in situ have demonstrated that Ca^2+^, cAMP, IP_3_ and DAG do not diffuse isotopically; rather, they move through rheologic “corridors,” i.e., areas of decreased mechanical resistance created by aligned polymers or fluidization dependent upon ATP [82]. This creates a computational property analogous to reaction–diffusion anisotropy that permits local biochemical circuits to operate in a directionally sensitive manner and take into account their environment [83].

The movement of mitochondria, lysosomes and endosomes in non-uniform environments is influenced by the local rheologic state. Additionally, mitochondrial movement has viscosity-dependent stalling thresholds which create “energy shadows”—areas in which energy delivery to the mitochondria is delayed due to the viscous drag associated with the local rheologic state. These energy shadows influence synaptic fatigue, the rate of dendrite recovery, and the susceptibility to metabolic stress [84]. Additional recent data demonstrates that intracellular water layers undergo the depolarization-induced reorganization of the water layers in the cell, changing between more and less ordered hydrogen bonding networks. These hydration state transitions affect proton mobility, the dielectric constant, and the thermodynamics of enzymatic catalysis. The proton hopping rates along hydrogen-bonded chains near membranes and cytoskeletal surfaces are also believed to modulate redox reactions and reactive oxygen species’ (ROS’) microdomains. These microdomains, in turn, produce transient viscosity changes through the oxidation of cytoskeletal proteins, and thereby couple redox fluctuations to rheologic states [85,86].

Thus, the cytoplasmic rheology acts as an adaptive filter, influencing the timing, magnitude and extent of the signal transmission within the cell through non-equilibrium physical processes rather than simply through the kinetic properties of enzymes [87].

### 5.2. LLPS in Synaptic and Extrasynaptic Domains: Phase State Encoding of Activity History

Phase separation enables neurons to organize complex biochemical systems in non-membrane-bound domains that reflect the physical state of the domain, and therefore encode the history of the activity of the neuron. At the presynaptic terminal, the phase-separated scaffolding formed by RIM, RIM-BP and ELKS exhibits phase hysteresis. Once formed, these condensates will remain in a partially fluidized state even when electrical activity ceases. Therefore, these structures provide a mechanism for preserving functional context in the absence of protein synthesis [88].

Similarly, postsynaptic densities also exhibit phase behavior. High-resolution imaging indicates that the PSD condensates undergo microphase partitioning, resulting in multiple nanoscale clusters with different viscosities and molecular compositions. During LTP, certain sub-modules of the PSD undergo phase compaction while other sub-modules remain in a fluid state to allow for the rapid exchange of receptors [89]. Thus, PSDs can simultaneously tune the stability and flexibility of the synapse. Evidence is emerging that phase separation occurs outside of synaptic regions as well. Signaling hubs formed by proteins such as CaMKIIδ, Kv2.1 clusters, and Eph receptor assemblies also exhibit condensate-like behavior during sustained electrical activity. These signaling hubs function as buffer zones that sequester kinases, phosphatases and lipids to smooth out fluctuations in signaling throughout the dendritic shaft [76].

Finally, phase separation plays a role in maintaining the molecular architecture of the axon initial segment (AIS). The AIS is responsible for coordinating the generation of the action potential, and appears to do so in part by maintaining condensates formed by ankyrin-G, βIV-spectrin, and CAMs under high local ionic oscillations. The electrical state influences the properties of the condensates in this region by modulating the Ca^2+^-dependent phosphorylation of intrinsically disordered protein segments. RNA metabolism also undergoes phase regulation in response to electrical activity [90]. RNP granules undergo frequency-dependent phase transitions. Low-frequency depolarization promotes liquid-like states that facilitate mRNA transport, while high-frequency bursts induce gel-like states that stabilize translationally silent pools. This creates a frequency-to-phase transformation, relating the regime of electrical activity to the availability of translation. Together, these findings establish LLPS as an activity-sensitive material code that bridges electrical patterns and spatially organized biochemical states [91].

Conceptually, the processes of LLPS can be thought of as a processing operation. The functions of this operation include (1) bringing together particular combinations of molecules to produce regions of space or “micro-reactors” that will vary both locally in chemical potential and locally in reaction rates, (2) allowing the system’s dependency on the past history of events through the presence of hysteresis and variable viscosity, (3) providing for the simultaneous optimization of both the stability and plasticity of the PSD microphase separation into regions of differing physical characteristics, and (4) converting the frequency of electrical activity into material states (gel-like vs. liquid-like), thereby affecting both trafficking and translationally accessible states [92].

Additionally, it should be noted that the capability of performing each of these functions as part of LLPS depends on the ability of the electromagnetic force to modulate the physical properties of the condensates themselves; i.e., the electrical activity and Ca^2+^-dependent phosphorylation can cause changes in the properties of the condensate at locations, including the AIS, while the dielectric and viscous states of the condensate can affect the channel mobility and access to kinases/phosphatases on timescales consistent with behavioral expression. Thus, LLPS represents a physiochemically based mechanism to relate rapidly occurring patterns of electrical activity to slower, spatially organized biochemical states which function as a stability mechanism for computations that do not need to occur within previously determined membrane boundaries [93].

### 5.3. Soft Matter Interactions at Organelle Interfaces: Phase-Modulated Nanojunctions and Quantum–Hydration Feedback

In addition to functioning as biochemical exchange zones, organelle contact sites in neurons function as soft matter interfaces whose physical state influences the fidelity of signaling. ER–mitochondria contact sites, for example, exhibit liquid-like behavior when ATP is plentiful and gel-like behavior when the cell is experiencing metabolic stress. These transitions change the intermembrane space and, in doing so, change Ca^2+^ flux, lipid transfer, and ROS buffering [94].

Recently identified findings demonstrate that nanojunctions undergo mechanically assisted phase switching. Tension exerted by the cytoskeleton can increase the width of ER–mitochondria gaps by a few nanometers, which reduces the amplitude of Ca^2+^ microdomains and alters the membrane potential of the mitochondria. Conversely, depolarization-induced localized acidification or ROS bursts can cause the formation of condensates at the nanojunctions, increasing the density of tethers through LLPS of VAPB–PTPIP51 complexes. In addition to classical biophysics, preliminary findings also suggest novel quantum–hydration interactions occur at these interfaces [95]. The tunneling events of protons through structured water networks at ER–mitochondria contact sites may influence the local redox potential, which in turn modulates the phase behavior of lipid transfer protein complexes. While speculative, these mechanisms illustrate how phase state transitions at nanojunctions could integrate both chemical and physical variables into signaling architectures [96].

Contact sites between lysosomes and mitochondria also exhibit dynamic behavior. Transient condensates formed by the Rab7–FKBP1 family of effectors regulate mitochondrial fission and mitophagy initiation. These condensates rapidly dissolve under increases in cytosolic ATP or pH changes, establishing a link between metabolic rhythms and organelle quality control [97].

**Figure 2:**
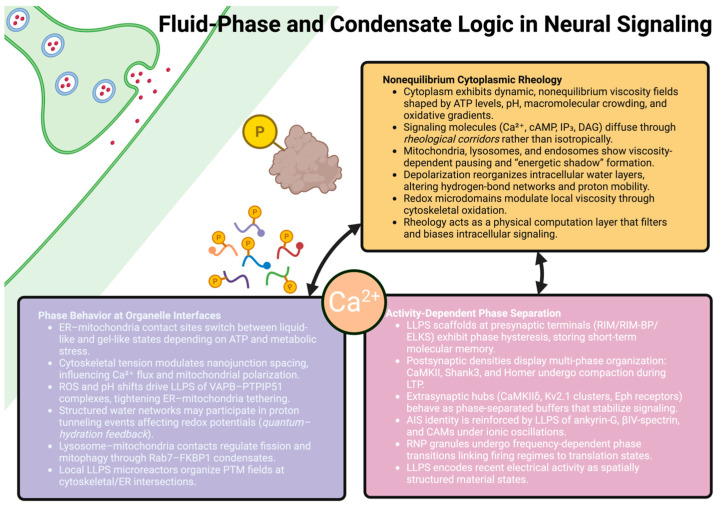
Depicts how the non-equilibrium rheological properties of cytoplasm, the activity-dependent condensation of cellular components, and the phase-dependent interactions between organelles regulate signal flow at the Intracellular level in neurons.

Lastly, transient liquid-like assemblies—termed micro-reactors—exist in small numbers along axons and dendrites that coordinate the local post-translational modification of proteins. For example, the LLPS of calmodulin-binding proteins at microtubule–ER interfaces shapes the phosphorylation fields that modulate microtubule stability in real-time. Taken collectively, the dynamics of organelle interfaces generate a phase state scaffold that integrates mechanical deformation, ionic oscillations, viscosity changes and redox fluctuations into coherent patterns of intracellular signaling. These nanojunctions represent central computational nodes in the cell where biophysical constraints converge [98]. Figure 2 attempts to summarize how neurons utilize fluid phase dynamics and condensate behavior to organize the flow of intracellular signaling.

## 6. A Unified Framework for Electromechanical–Phase Computation

### 6.1. Cross-Domain Coupling Through Shared Physical Interfaces

Shared molecular interfaces provide a basis for the cross-coupling of electrical, mechanical and fluid phase processes, which are all interconnected physically. As these interfaces are subject to dynamic changes due to their ongoing activity, this cross-coupling provides a basis for the communication between the domains. The interfaces include lipid–protein complexes, mechanosensitive scaffolds, cytoskeletal anchoring hubs and phase-regulated protein assemblies [99]. These interfaces function as bridges between the different physical domains, allowing perturbations of one domain to induce responses in the other domains. New imaging techniques have demonstrated that small electrical perturbations can cause significant local curvature changes in the membrane by causing the redistribution of charge within lipid headgroups and changing the intermolecular forces between them [100]. This curvature change will affect the mechanical tension on tethered cytoskeletal proteins such as ankyrin-G, βIV-spectrin and ERM-family linkers, and therefore the mechanical tension at the nanometer scale away from the membrane. Perturbations in mechanical tension will then be transmitted into the cytoplasm where it will alter the threshold for the condensation of proteins containing intrinsically disordered regions, and therefore it will alter the formation or dissolution of liquid-like assemblies [31].

On the other hand, phase transition will affect electrical behavior. Liquid-like assemblies rich in charged RNA-binding proteins will change the dielectric environment and reduce the lateral diffusion of voltage-gated channels and therefore the current flow along the membrane. Changes in the viscosity of condensates will change the accessibility of kinases and phosphatases that will control the phosphorylation status of the ion channels and therefore the activation threshold on millisecond to second timescales [101]. Therefore, these interfaces will represent the two-way conversion between the electrical signals, mechanical deformations, and phase transitions. Therefore, the integration of these interfaces represents a continuously updated multiphysics architecture that can support information exchange beyond the exchange of ions alone [102].

### 6.2. Multiscale Integration and Emergent Attractor States

In contrast to each of the electrical, mechanical and fluid phase systems being examined separately, the cross-coupling between these systems creates emergent behaviors that cannot be explained by analyzing each system separately. At the nanoscale, millisecond-long precise changes in ion channel gating, membrane bending, or condensate fluidity create localized chemical and mechanical environments within single synapses or dendritic segments [103].

At the micron scale, these rapid perturbations create structural–functional attractor states—stable associations between mechanical stiffness, condensate composition, and electrical responsiveness. For example, a dendritic region that has been repeatedly activated by synaptic input can develop into an attractor state that includes specific spine stiffness, viscosity minima in localized areas, and the persistent clustering of ion channels. These attractors contain the history of past activity as a result of the physical organization of the attractor, and provide a form of biophysical memory that is independent of synaptic weight changes [104,105].

At the mesoscale, groups of neurons show network-level coherence, which is partly based on mechanical and phase state variables. Mechanical coupling through extracellular matrix fibers or astrocyte processes affects the synchronization of action potentials through the modification of conductance properties and transmembrane tension. Fluid phase dynamics control the spread of second messengers among connected compartments, and influence the timing of synchronized activity and the emergence of oscillatory states [106]. Simulations of neural networks using new methods that incorporate multiphysics constraints indicate that neural networks with electromechanical–phase coupling have a wider range of computational capabilities, including the ability to create slow manifolds, increased sensitivity to subthreshold inputs and robust state transitions in the presence of noise. These characteristics emerge from the interaction of processes with very different relaxation times and enable the integration of information over many different timescales (milliseconds, seconds, etc.) [107].

### 6.3. Thermodynamic and Energetic Structure of the Electromechanical–Phase System

The electromechanical–phase connectome functions as a dissipative, non-equilibrium system supported by continuous energy flow from ATP hydrolysis, ion gradient dissipation, redox reactions and cytoskeletal polymerization. Its computational capability arises from how the energy available in one modality is allocated to another:

- Energy generated by electrical processes is quickly dissipated through ionic currents and generates transient, sharp perturbations [108].
- Energy stored in mechanical processes is redistributed by the elastic deformation of membranes, filaments and scaffold proteins [109].
- Persistent energetic minima are created through phase state processes, such as condensate formation, viscoelastic transitions and changes in hydration structures [110].

There are energetic layers that interact with each other through coupled order parameters. Electrical perturbations modify the potential energy of the membrane, mechanical deformation modifies the elastic energy landscape, and phase transitions modify the free energy through the reduction in configurational entropy. Therefore, the order parameters are evolved in link to trajectories and provide energy transfer pathways that allow the neuron to stabilize or destabilize a particular functional state [111].

Recent calorimetric studies indicate that phase transitions in condensates generate measurable enthalpic signatures, which indicate that they behave as intracellular thermodynamic buffers during prolonged activity. During prolonged activity, condensates may capture transient energetic fluctuations and preserve signal fidelity by stabilizing reaction kinetics [112]. Elastic reservoirs such as actin networks also distribute energy over segments of the cell. These energy handling mechanisms allow the neuron to create metastable states at multiple scales—states that are not fixed attractors but remain stable for sufficient time to influence computation. The metastability provides a mechanism that allows the neuron to avoid chaotic dynamics and still maintain flexibility, which is a feature that emerges specifically from the integration of electrical, mechanical and soft matter processes [113].

Collectively, these thermodynamic concepts present the electromechanical–phase system as a comprehensive framework of computation where information is encoded in both the electrical patterns and in the distributed physical states that are formed by the movement of energy and by material constraints [114]. Section 6 integrates electrical, mechanical and fluid phase processes into a common model of computation. In addition, Section 6 presents the concept of cross-domain coupling through shared molecular interfaces, describes how multiphysics interactions create multiscale attractor states, and presents the electromechanical–phase dynamics in thermodynamic terms. These principles provide a conceptual basis for understanding a computational architecture that relies on integrated physical systems, rather than on isolated biochemical pathways.

In addition to advances in understandings of neuro-mechanical dynamics in biological systems, there is increasing evidence of progress in the development of artificial systems capable of simulating some aspects of this process. Specifically, in recent years, there has been a significant amount of work in developing “neural networks” using artificial synaptic elements based on memristive devices, i.e., devices that are capable of changing their resistance in a manner that is dependent upon prior history [115]. These studies represent a type of engineered embodiment of a basic premise of the proposed electromechanical–phase connectome, namely that memory and computation can be realized with history-dependent state variables that are non-electrical, but which rely on cooperative reorganizations at the ionic, structural and phase levels [116].

Aspects of the behavior of memristive devices have been explored in relation to their use as “synaptic” elements in both individual devices and as components of larger networks of devices (e.g., crossbar arrays). Conductance changes in these devices result from the field-dependent motion of ions, redox reactions, filament formation/dissolution, joule heating, or phase transitions, depending on the specific family of device being used. When implemented in a network, such as a crossbar array or in a neuromorphic architecture, the resulting response of the system to repetitive stimulation demonstrates convergent behavior that reflects stimulus-selective response properties, similar to those seen in biological systems [117]. Consequently, these systems demonstrate examples of how multistable states may arise from the dissipative processes associated with the energy required to drive the system and the constraints imposed by the material composition of the devices. While the devices themselves are synthetic, the physical principles governing their behavior provide an analogical relationship to the current conceptual framework in that they demonstrate how electrical input may induce a change in the physical substrate that stores a state, biases subsequent trajectories, and enables robust pattern completion [118]. As a result, memristiv–synapse coupled networks may be viewed as a controlled, simple version of multiphysical computing and therefore provide additional direction for translation towards the experimental validation of the implications of state-variable-constrained attractor models of neural stability and degeneration [119].

## 7. Pathological Disruption of the Electromechanical–Phase Connectome

### 7.1. Electrical Instability Originating from Mechanical and Phase-State Perturbations

Numerous neurological disorders arise from variations in physical states, prior to biochemical or structural deviations being evident. The oxidative modification of polyunsaturated fatty acids (a hallmark of early neurodegenerative disease) alters lateral pressure profiles in biological membranes and alters the normal voltage-sensing energetics of Na^+^, K^+^ and Ca^2+^ channels [120]. These changes alter the kinetic gating, the current–voltage relationships and introduce abnormal subthreshold oscillations that compromise spike timing before any synaptic loss occurs [121].

Mechanical failure provides an additional dimension of electrical instability. Disruption of the axonal spectrin–actin lattice (observed in traumatic injury, metabolic compromise and with age) results in heterogenous membrane tension and the localization of Nav1-family channels to inappropriate locations on the plasma membrane [122]. Spike jitter, intermittent conduction failures and the reduced fidelity of high-frequency transmission are generated. Weak connections between the nucleus and cytoskeleton also alter tension across the nuclear envelope and impede activity-dependent chromatin remodeling, resulting in irregular transcriptional responses to electrical stimuli [123].

Electrical instability can be produced by physical state disruptions via disorder, rather than the loss of channel proteins. Hardened or fragmented condensates in the AIS disrupt the necessary nanoarchitecture for the clustering of voltage-gated channels. Pathological changes in the self-assembled LLPS of scaffolding proteins at the postsynaptic density lead to non-aligned postsynaptic nanodomains and non-coincident pre- and postsynaptic geometry, thereby reducing synaptic precision, even if ionic currents are preserved. These physical irregularities prior to the development of classical biochemical markers represent a class of “pre-electrical” pathologies that modify excitability long before classical biochemical markers have appeared [124].

### 7.2. Mechanical Failure Modes and Subcellular Architectural Breakdown

Neuronal geometry and signal propagation are critically dependent upon maintaining mechanical homeostasis. In tauopathies, excitotoxic conditions and in mitochondrial dysfunction, microtubules lose their stability and elastic properties, thereby causing localized rigidification or the collapse of segments of dendrites and axons, preventing organelle transport and establishing metabolically deprived zones. The isolation of portions of a neuron from tension-based signaling occurs as mechanical coherence deteriorates; therefore, portions of a neuron can no longer contribute to the neuron’s overall response to input [125]. In addition to the failure of mechanical homeostasis, the failure of curvature-regulating machinery contributes to additional pathology. The reduced activity of BAR-domain proteins (reported in models of aging, metabolic disease and chronic inflammation) reduces the membrane curvature that is necessary for the formation of synaptic vesicles and the maturation of endocytic pits. As the ability of a cell to generate curvature decreases, the number of cycles of vesicle release and retrieval that can occur in the presynaptic terminal decreases, and therefore creates a functional bottleneck [126].

The instability of mitochondrial shape represents yet another mechanically based pathology. The fragmentation or swelling of mitochondria causes changes in the stiffness of the organelle and its ability to withstand compression from the surrounding cytoskeleton. These physical changes disrupt the geometry of the contacts between the ER and mitochondria and create irregular calcium microdomains, which compromise ATP production and increase the vulnerability to excitotoxic stress. Therefore, mechanical failure serves not only as a secondary consequence of cellular damage, but as a primary destabilizing factor that alters the viability of neurons at subcellular resolution [127].

### 7.3. Breakdown of Soft Matter Homeostasis: Aberrant Condensation, Viscosity Shifts, and Phase Pathology

Disorders of phase behavior constitute a wide variety of neuropathology. In ALS, FTD, and certain types of AD, mutations in low-complexity domains cause the condensates to transition to a gel-like or solid-like phase. These altered phase boundaries limit the dynamics of ribonucleoprotein granules, trap essential mRNAs, inhibit local translation, and create intracellular viscosity gradients that prevent the free diffusion of signaling molecules. These gradients disrupt calcium buffering, metabolic delivery, and proteasomal flux and therefore create uneven cellular stress. Additional pathology is created by the impaired turnover of condensates [128]. For lysosomes and proteasomes to enter and degrade aggregated assemblies, they must be able to penetrate and access condensates of specific viscosity. If the condensates become hardened, this will stop these clearance processes and initiate a feedback loop of accumulation, compaction and metabolic exhaustion [129].

The disintegration of domain coordination may result from progressive deterioration of coordination among three essential attributes of neuronal function: electrical precision, mechanical integrity, and the maintenance of homeostasis of the phase state. The disruption of these three critical functions can lead to the loss of the stability of the system, with the disruption typically preceding the clinical symptoms of neurodegenerative disease through biomarkers of neuronal degeneration [130]. Thus, the perspective presented here identifies additional, potentially novel therapeutic strategies that stabilize those physical parameters of neuronal function contributing to computation failure in neurodegenerative disease [131]. Some examples of how neural function may be stabilized during its breakdown include the following: the preservation of membranes’ electromechanical properties through limiting oxidative lipid remodeling, which alters the lateral pressure profile and energy associated with voltage sensing; the conservation of axonal and synaptic mechanical coherence by maintaining structural organization of spectrin–actin lattices, and the elastic stability of microtubules and the machinery generating the curvature necessary for vesicle cycling; the restoration of the geometry and mechanochemical resistance of interfaces between organelles (e.g., ER–mitochondrial contact sites) to reduce pathological oscillations in Ca^2+^ levels and metabolic susceptibility; and the stabilization of the homeostatic equilibrium of soft materials through prevention of the maladaptive transition from the liquid to solid/gel phases of condensates to prevent pathological consequences resulting from diminished diffusion and buffering through viscosity gradients and the enhanced access of proteostases to assemblies that cannot undergo lysosomal or proteasomal degradation because of their material properties [132]. Although there are numerous types of therapies directed at the aforementioned targets, they all share the common goal of either conserving or restoring the electromechanical–phase structure of neurons to maintain an attractor-like steady-state function and thereby to prolong the time it takes for the “computation collapse” of neural systems to occur [133].

Biological soft matter dysregulation can affect membrane-associated structures as well. In Parkinsonian conditions, α-synuclein transitions from a dynamic membrane-bound ensemble to a more viscous assembly that alters membrane curvature and reduces the efficiency of vesicle fusion. Similarly, pathological condensates at ER–mitochondria junctions disrupt lipid exchange and calcium transfer, and therefore disrupt intracellular homeostasis. Therefore, phase state pathology can propagate through many levels of the electromechanical–phase connectome and can alter both mechanical integrity and electrical responsiveness, and metabolic stability simultaneously [134].

### 7.4. Network-Level Degradation and Computational Collapse

As electromechanical–phase disruptions continue to develop, circuit-level function continues to deteriorate even though there may be little or no apparent cell loss. Mechanical fragility in axons produces irregular signal propagation and fragments communication along long-range pathways. The changes in astrocytes, particularly the increase in viscosity in fine perisynaptic processes, interfere with potassium buffering, glutamate removal and extracellular volume regulation, creating slow fluctuations in the ionic landscape that degrade high-frequency computation [135].

Abnormal cytokine release and irregular patterns of extracellular matrix remodeling caused by phase instability in microglia produce abnormal mechanical properties of the tissue and disrupt mechano-electrical interactions throughout the circuits. As circuit nodes lose mechanical and phase state coherence, the stability of oscillatory patterns decreases, the depth of attractor states decrease and the transitions between network states become erratic [136].

Finally, the converging multiphysics vulnerabilities cause a type of computational collapse in which a neuron and its surrounding network fail to maintain the physical stability required to reliably process information. Thus, neurodegenerative disease arises from the gradual disassembly of the electromechanical–phase architecture that supports neural computation [137]. Neuropathological conditions destroy neuronal function by destroying electrical precision, mechanical integrity, and phase state homeostasis. Diseased states result from the gradual loss of physical coordination among these domains, ultimately leading to the multi-scale failure of signaling, structure and computation.

## 8. Conclusions: The Beginning of a Physics of Thought

Historically, neuroscience has explored the brain in terms of electricity, chemistry, and synapses. Each has provided substantial contributions to our knowledge base. However, it is evident from the data collected here that the mechanisms underlying these disciplines provide only one dimension of an expansive universe of possibilities. Neurons are not just electrically active devices, but rather are multiphysical systems that exhibit an evolving interaction of membrane curvature, molecular tension, cytoplasmic rheology, intracellular fluid movement, soft matter transitions, and voltage-induced structural changes.

In this expanded scope, electrical activity is simply one manifestation of a deeper organization based upon physical principles. Voltage can modify the structure of membranes, mechanical stress can rearrange cytoskeletal structures, phase transitions can alter biochemical accessibilities, and fluid phase constraints can establish spatially constrained diffusion pathways directing molecular movement. Each of these domains does not occur in a linear progression but instead interacts dynamically to create a constantly evolving architectural framework in which information is encoded simultaneously within ionic fluxes, mechanical states, and phase behaviors.

A comprehensive perspective of the neuron as a dissipative material system has become apparent. Neurons can store energy within elastic networks, distribute mechanical forces throughout periodic cytoskeletal lattice structures, encode memory within condensate architectures, and stabilize computation via viscosity gradients evolving over timescales that are many orders of magnitude larger than the durations of action potential firing. This type of architecture enables neurons to compute using multiple physical substrates concurrently, each contributing their respective temporal logic and stability characteristics.

Disorders of the nervous system, viewed from this perspective, represent disruptions of this organized physical framework—homeostatic failures of tension, curvature maintenance, viscosity, organelle tethers, or phase boundary regulation. Therefore, the electrical symptoms observed in many disorders often reflect the ultimate manifestations of a collapse of electromechanical–phase coherence. The technologies necessary to map out this expanded framework are only now emerging. High-speed cryogenic tomography can demonstrate the nanomechanics of spine head structures, interferometric probes can detect force propagation through the dendritic membrane, rheological sensors can detect fluidization events occurring as a result of rapid bursts of activity, and computational models combining soft matter physics and electrophysiology are starting to elucidate behaviors that cannot be predicted by traditional modeling approaches. Collectively, these emerging technologies indicate a future where the physicality of the living neuron can be characterized with the same precision used to characterize its electrical properties.

The objective of this review was not to present a complete theory, but to outline a path that the field is already traveling—a path where electrical, mechanical, and phase state processes are considered the coordinated variables of a unified computational framework. A path where the physical aspects of the neuron—its tension, its viscosity, its thermodynamic posture—will be equally important in the explanation of cognition as the flow of ions. There are many uncertainties remaining in this new landscape. The distinctions between electrical, mechanical, and soft matter processes are not absolute; there is a gradual and nonlinear transition between these processes. Their collective dynamics may support forms of computation that have not been fully conceptualized. The field currently exists at the threshold of this new landscape, with the most significant studies being yet to come.

If the ideas presented here inspire further research into the physicality of neural functions—if they motivate a greater focus on the physical attributes that affect signaling, plasticity, and susceptibility—then the purposes of this manuscript will have been fulfilled because the story of the electromechanical–phase connectome is not complete; it is only beginning and will probably significantly redefine the way we understand the nervous system at its most basic levels.

## Figures and Tables

**Table 1:** Describes the major biophysical components that form the basis for the electromechanical connectome. In addition, it describes how voltage sensing (energetics), cell mechanics (cytoskeleton and membranes) and intracellular rheology, along with phase state control all operate at different scales to determine the neuronal excitability, signal transmission and adaptative computations.

Biophysical Tier	Core Mechanisms and Molecular Players	Electromechanical Consequences for Neural Signaling	Experimental/Computational Correlates	References
Voltage sensing and gating landscapes	Hierarchical S4 voltage sensor transitions across multiple metastable states; gating charge displacement shaped by lipid headgroups, membrane dipoles, and hydration shells rather than pure helix translation alone.	Fine-tuning of activation/inactivation thresholds; isoform-specific gating heterogeneity across axon initial segment, dendritic shaft, and presynaptic boutons; and dynamic reshaping of excitability with small changes in local field and solvent structure.	Time-resolved cryo-EM of intermediate VSD conformers; atomistic MD of channel–lipid–water complexes; and gating current spectroscopy resolving multistep charge movement.	[40]
Lipid electrodynamics and hydration shells	PIP_2_, phosphatidylserine, and diacylglycerol create anisotropic lateral electric fields; ultrafast restructuring of interfacial water modulates local dielectric constant and proton mobility at the protein–lipid boundary.	Local shifts in effective gating charge and barrier heights; region-specific tuning of channel kinetics and voltage dependence; and emergence of microdomain-specific operating modes from a single channel isoform.	Ultrafast IR and 2D-IR spectroscopy of hydration dynamics; MD/continuum hybrid electrostatics; and voltage-clamp recordings under controlled lipid reconstitution.	[41]
Ionic nanodomains and electro-osmotic microgradients	Cav2.1/2.2 Ca^2+^ nanodomains (10–50 nm) with steep concentration gradients; K^+^-efflux microdomains generating osmotic and tension shifts; and transient surface potentials modifying neighboring channel open probability.	Highly localized activation of vesicle fusion and Ca^2+^-sensitive enzymes; spatially patterned electrochemical fields beyond resolution of classical electrophysiology; and coupling of ion flux to water flow and membrane mechanics.	Ca^2+^ nanodomain mapping with fast indicators and buffers; stochastic channel simulations; and imaging of osmotically driven membrane deformation.	[42]
Cytoskeletal force networks (actin, microtubules, spectrin)	Actin–myosin contractility and actin–cofilin stiffness cycling on sub-second timescales; microtubule stiffness set by nucleotide state and PTMs (polyglutamylation, detyrosination); and axonal αII–βII spectrin–ankyrin lattices acting as periodic elastic springs.	Redistribution of mechanical load across axon and dendrites; mechano-tuning of Na_v_ clustering and firing threshold; preservation of axolemmal integrity during high-frequency activity; and anisotropic propagation of deformation signals.	Single-molecule force spectroscopy on actin and MTs; AFM mapping of axonal spectrin elasticity; and live cell imaging of deformation propagation along cytoskeleton.	[43]
Adhesion complexes and curvature sensors	Integrin–talin–vinculin mechanotransduction units unfolding under load and exposing cryptic binding sites; cadherin-based junctions transmitting tensile cues; and BAR/F-BAR/I-BAR proteins recruited to curved membranes converting geometry into signaling.	Translation of extracellular stiffness, shear, and curvature into cytoskeletal remodeling and channel localization; curvature-dependent control of endocytosis, spine remodeling, and presynaptic vesicle cycling.	Optical tweezers and traction-force microscopy of adhesion complexes; super-resolution imaging of BAR-domain localization; and curvature-controlled nanotube and vesicle assays.	[44]
Cytoplasmic rheology and viscoelastic constraints	Viscoelastic cytoplasm with ms–s relaxation spectra governed by polymer entanglement, transient binding, and organelle crowding; spatial gradients of effective viscosity along neurites.	Compartment-specific diffusion times for proteins, metabolites, and second messengers; shaping of Ca^2+^ wave spread, kinase cascades, and signal integration timescales; and modulation of coupling between membrane events and nuclear responses.	Active microrheology and optical trapping; particle-tracking velocimetry; and coarse-grained simulations of viscoelastic cytoplasm.	[12]
Phase-separated condensates and soft-matter reaction crucibles	LLPS of PSD-95, SynGAP, RIM, FUS and related IDR-rich proteins into condensates with tunable viscosity, mesh size, and interfacial tension; aging-dependent transitions from liquid-like to gel-/solid-like states under stress.	Local amplification or damping of signaling via concentration, residency time, and turnover control; creation of micro-reactors that set thresholds for synaptic plasticity, stress granule dynamics, and transcriptional responses.	FRAP, single-molecule tracking, and rheology of condensates; in vitro reconstitution of synaptic and RNP droplets; polymer physics and sticker–spacer modeling.	[45]
Hydration layers, proton transfer and phase boundaries	Structured water at interfaces with lipids, filaments, and condensates alters local dielectric landscape, ion mobility, and proton transfer pathways; osmotic and ionic changes shift condensate phase boundaries (fusion, fission, dissolution).	Fine control of enzyme kinetics, complex stability, and phase transition likelihood; coupling of metabolic state and osmolarity to reconfiguration of signaling landscapes and organelle access.	Ultrafast spectroscopy of interfacial water; QM/MD simulations of proton transfer; and phase diagrams of condensates under ionic/osmotic perturbation.	[46]

**Table 2:** Outlines the unique computational processes introduced into different regions of neurons through the application of distinct mechanical forces. This table describes how molecules that sense mechanical force (from mechanosensitive channels to cytoskeletal lattice proteins to presynaptic scaffold proteins) interpret three physical properties of mechanical forces—tension, curvature, stiffness, and viscoelasticity—and how this interpretation influences excitability, synaptic transmission efficacy, vesicle movement, and signal fidelity.

Computational Domain	Mechanical Inputs	Molecular/Structural Transducers	Resulting Computational Operation	Functional Consequences for Neural Signaling
Mechanosensitive Gating (PIEZO1/2, K2P, ASICs, and TRPV4)	Membrane tension, curvature, osmotic strain, and lipid lateral pressure	Curved PIEZO blades; K2P pressure-sensitive helices; ASIC protonation landscapes; and TRPV4 lipid-dependent allostery	Tension-encoded conductance states; graded depolarization/hyperpolarization; and microdomain-level excitability logic	Rapid mechanical → electrical transduction; stabilization of firing thresholds; and spatially patterned excitability maps
Lipid–Protein Coupling in Ion Channel Modulation	Bilayer pressure asymmetry; acyl-chain heterogeneity; and curvature stress	PIP_2_, DAG, and PS microdomains; curvature-sensitive lipids; and mechanosensitive annular lipid shells	Shifted gating charge energetics; altered transition barriers; and local tuning of open/inactivated states	Region-specific channel behavior (AIS vs. dendrite vs. bouton); enhanced computational diversity from identical channel isoforms
Spine Head Mechanics	Synaptic deformation; actin turnover; and localized bending	Actin–cofilin complexes; tropomyosin patterns; and spectrin crosslinking	Mechanical shaping of ionic microgeometry; stiffness-dependent filtering of EPSPs	Mechanical gain control; modulation of voltage transfer to dendrite; and refined synaptic weight tuning
Receptor Field Reorganization	Membrane curvature pulses; local bending gradients	Endophilin and amphiphysin; curvature-sensing BAR proteins	Mechanically driven AMPAR clustering; lipid defect-guided receptor insertion	Enhanced synaptic fidelity; precise alignment with presynaptic release zones
Mechanochemical Signaling Within Spines	Strain on phosphoinositide-rich domains; local tension shifts	PIP_2_ and PIP_3_ accessibility changes; PLCβ and PI3K mechanosensitivity	Stiffness-linked modulation of DAG/PIP_3_ production; tension-defined biochemical states	Spatially restricted signaling; ultrasensitive to mechanical compliance
Dendritic Shaft Mechanics	Branch curvature; cytoskeletal anisotropy; and load distribution	MAP-dependent microtubule bundling; actin–microtubule crosstalk	Direction-dependent signal attenuation; mechanical filtering of integrated inputs	Analog computation governed by morphology and mechanical gradients
Presynaptic Tension Fields	Active zone deformation; scaffold elasticity	ELKS–RIM–Munc13 networks; tensioned docking sites	Curvature-dependent vesicle priming; SNARE zippering enhancement/suppression	Release probability heterogeneity, microdomain precision in vesicle fusion
Actin–Synapsin Matrix Mechanics	Activity-induced softening or stiffening	Synapsin elasticity; actin viscoelasticity	Mechanical gating of vesicle mobility and RRP replenishment	Control of sustained firing capacity; fatigue vs. facilitation dynamics
Endocytosis Under Mechanical Constraint	Membrane tension; bending energy; and local curvature reservoirs	BAR-domain proteins; dynamin mechanochemical constriction	Tension-coupled pit formation and scission; mechanically matched retrieval rates	Maintained vesicle supply; coupling of exocytosis with mechanical recovery
Axonal Spectrin Lattice Mechanics	Longitudinal strain; firing-associated deformation	αII–βII spectrin periodic scaffolds; ankyrin-G anchoring	Strain distribution and recoil dynamics; maintained nodal geometry	Fidelity of action potential conduction; mechanical safeguarding of presynaptic operation

## Data Availability

No new data were created or analyzed in this study. Data sharing is not applicable to this article.
